# Association Between Acute Kidney Injury Hospital Visits and Environmental Heat Stress at a Nicaraguan Sugarcane Plantation

**DOI:** 10.1177/21650799241235410

**Published:** 2024-04-09

**Authors:** Erik Hansson, Kristina Jakobsson, Jason R. Glaser, Catharina Wesseling, Denis Chavarría, Rebekah A. I. Lucas, David H. Wegman

**Affiliations:** 1 La Isla Network, Washington, District of Columbia, USA; 2 Occupational and Environmental Medicine, School of Public Health and Community Medicine, Sahlgrenska Academy, University of Gothenburg, Gothenburg, Sweden; 3 Occupational and Environmental Medicine, Sahlgrenska University Hospital, Gothenburg, Sweden; 4 Unit of Occupational Medicine, Institute of Environmental Medicine, Karolinska Institute, Stockholm, Sweden; 5 Occupational Health Management, Ingenio San Antonio/Nicaragua Sugar Estates Limited, Chichigalpa, Nicaragua; 6 School of Sport, Exercise and Rehabilitation Sciences, University of Birmingham, Birmingham, UK; 7 University of Massachusetts Lowell, Lowell, Massachusetts, USA

**Keywords:** injury/occupational injury/traumatic injury, global occupational health, epidemiology, occupational health and safety programs, chronic illnesses

## Abstract

**Background:**

Mesoamerican sugarcane cutters are at a high risk of chronic kidney disease of non-traditional origin, a disease likely linked to heat-related acute kidney injury (AKI). Studies in general populations have described a positive association between high environmental temperatures and clinically assessed kidney outcomes, but there are no studies in occupational settings.

**Method:**

We accessed routine records of clinically diagnosed AKI (AKI-CD) and wet bulb globe temperatures (WBGT) at a large Nicaraguan sugarcane plantation and modeled the relationship between these using negative binomial regression. A rest-shade-hydration intervention was gradually enhanced during the study period, and efforts were made to increase the referral of workers with suspected AKI to healthcare.

**Results:**

Each 1°C WBGT was associated with an 18% (95% confidence interval [CI]: [4, 33%]) higher AKI-CD rate on the same day and a 14% (95% CI [−5, 37%]) higher rate over a week. AKI-CD rates and severity, and time between symptoms onset and diagnosis decreased during the study period, that is, with increasing rest-shade-hydration intervention. Symptoms and biochemical signs of systemic inflammation were common among AKI-CD cases.

**Discussion:**

Occupational heat stress, resulting from heavy work in environmental heat, was associated with a higher rate of clinically diagnosed AKI in a population at risk of CKDnt. Promoting rest-shade-hydration may have contributed to reducing AKI rates during the study period. Occupational health and safety personnel have key roles to play in enforcing rest, shade, and hydration practices, referring workers with suspected AKI to healthcare as well as collecting and analyzing the data needed to support workplace heat stress interventions.

## Introduction

Chronic kidney disease of non-traditional origin (CKDnt), that is, CKD not caused by traditional risk factors such as hypertension and diabetes, or other known causes (Pan American Health Organization, 2017), often affects heat-stressed Mesoamerican workers, especially sugarcane harvest workers (Wesseling et al., 2020). Repeated heat-related systemic inflammation combined with the physiological strain put on the kidneys when performing strenuous physical labor in the heat has been proposed as the pathophysiological mechanism underlying this disease (Hansson et al., 2020). Although there is at present no consensus on the etiology, occupational heat stress exposure is a key driver of disease (Wesseling et al., 2020).

Pronounced, acute elevations in kidney injury markers have been recorded in occupationally active sugarcane workers in Nicaragua (Hansson et al., 2019, 2021; Kupferman et al., 2018), El Salvador (Bodin et al., 2016; Wegman et al., 2018), and Guatemala (Butler-Dawson et al., 2019; Sorensen et al., 2019), as well as in sugarcane workers seeking care for a variety of symptoms (Fischer et al., 2017), and in heat stress laboratory studies (Chapman et al., 2020). Acute kidney injury (AKI) is considered to predispose workers toward developing CKDnt and/or having a more rapid reduction in kidney function (Fischer et al., 2018; Hansson et al., 2019; Kupferman et al., 2018), with AKI more common among sugarcane workers performing the most physically demanding jobs (Glaser et al., 2020; Hansson et al., 2019).

Occupational heat stress is determined by the combination of the worker’s metabolic heat production (i.e., the intensity of physical work) and the exchange of heat with the surrounding environment. The rate and direction of heat transfer between the body and environment is dependent not only on the ambient temperature, but also on the humidity and velocity of the surrounding air, solar radiation, and clothing. The wet bulb globe temperature (WBGT) is one of many metrics that aim to incorporate the environmental factors (air humidity and velocity, and solar radiation) determining the external heat stress into a single value. After finding that the adoption of a rest-work schedule based on WBGT successfully prevented heat illness in U.S. Army recruits in the 1950s (Yaglou & Minard, 1957), its use has become widespread, forming the basis of occupational heat stress prevention guidelines (Budd, 2008).

Studies in general populations have repeatedly reported an association between heatwaves and increased kidney disease morbidity (Liu et al., 2021). A recent meta-analysis found an overall 1% increase in the risk of various kidney diseases per 1°C ambient temperature in general populations, with a stronger effect in working-age men (Liu et al., 2021). Considering that higher environmental heat exposure and higher metabolic heat production than the general population can be expected in outdoor agricultural workers (Occupational Safety and Health Administration, 2022), this occupational group may be more sensitive to adverse kidney outcomes during hot days.

This study aims to describe the association between environmental heat and incidence of AKI at a hospital linked to a large sugarcane plantation in a Mesoamerican CKDnt hotspot. Second, we aim to describe how the incidence of hospital visits that led to a diagnosis of AKI has changed over 6 years of increased focus on the implementation of a workplace heat stress intervention. Finally, we aimed to explore whether healthcare-seeking patterns changed, in terms of patient delay, symptoms, and kidney injury severity.

## Methods

This is an occupational retrospective time-series analysis study from a sugar mill and its affiliated hospital which provides in- and outpatient care for workers and their families. The source population for this study includes all workers employed at Ingenio San Antonio (ISA), Nicaragua from January 2018 to May 2023. The study explores the association between environmental heat in the same and preceding days and the number of workers seeking hospital care for AKI. Within this analysis, we will also consider the long term trend in AKI hospital visits, which coincides with heat stress preventive measures being enhanced within the study population during the study period.

### Setting and Study Population

The sugarcane plantation, ISA, is located in one of the main CKDnt hotspots in Mesoamerica (Hansson et al., 2021). The tropical climate in the study area has a clear seasonal variation, with a dry season from November to April, and a wet season between May and October. Work at the sugarcane plantation follows a seasonal pattern, with the harvest of cane commencing in November. This is the time of the year when the workforce is markedly increased by fixed-term harvest workers who come from the surrounding communities, often returning year after year. No workers are migratory workers. The harvesting of mature cane, which is burned before harvesting, ends in April, while the green, living cane is harvested and used for planting from November to July. Currently, 5% of the cane is manually harvested, and the remaining 95% is harvested using mechanized harvesters. Other non-mechanized outdoor manual occupations at ISA include repair and management of irrigation tubes and canals, seeding, weeding, and pest control. The physical workload varies between these job groups, but is in many cases high, as previously described (Lucas et al., 2023).

Workers at ISA are screened before hiring, and those with suspected kidney dysfunction (serum creatinine >1.3 mg/dl for men, >1.0 for women in harvest 1 and harvest 2 [henceforth H1 and H2], thereafter estimated glomerular filtration rate [eGFR] <90 mL/min/1.73 m^2^) are retested and not hired if found to remain on the abnormal side of these thresholds.

### Occupational Health Interventions

Efforts to prevent heat stress have been in place at ISA for over 10 years (McClean et al., 2010) but were intensified and systematically evaluated via the Adelante Initiative (https://adelanteinitiative.org), which was followed by the PREP (Prevention Resilience Efficiency and Protection) research program. Within these research and intervention programs, the already existing heat prevention measures were observed in the 2017-2018 harvest (H1) in four of the main work groups (Hansson et al., 2019). Recommendations on improved work practices, by longer and more clearly enforced hourly Rest breaks and better access to Shade and Hydration (RSH; Glaser et al., 2020, 2022) were implemented for the 2018-2019 harvest (H2). Intervention implementation was then monitored and revised together with the mill occupational safety and health (OSH) department over that and the upcoming 2019-2023 harvests (H3-H6). This included a further increase in rest between H2 and H3 (Supplement Table 1) accompanied by ongoing reinforcement of the intervention protocol by the OSH department as necessary. For other workers at the mill, enhanced RSH interventions were also in place from H2, but the impact on kidney outcomes (Glaser et al., 2020) was not evaluated by the researchers, nor was intervention implementation as closely monitored by the researchers or OSH department. A qualitative organizational psychology assessment found that foremen throughout the mill were engaged in improving worker health (Pacheco-Zenteno et al., 2021). Thereby, although the Adelante Initiative and PREP research mostly focused on burned cane cutters, seed cutters, drip irrigation repair workers, and their accompanying supervisors and support staff (Glaser et al., 2020, 2022; Hansson et al., 2019) and later also on weeders, seeders and gravity irrigators (Lucas et al., 2023), these interventions and the lessons learned from them have benefited the mill’s workforce at large.

For many years, field workers at the mill have been followed by health promoters from the OSH department at the mill. Health promoters are trained nurses but receive further training by ISA on heat stress prevention, referral guidelines in case of suspected AKI, and management of minor workplace injuries. All manual agricultural work groups of approximately 60 workers are accompanied by a health promoter, who provides education, and monitors liquid intake and hydration outcomes. Cutters have in addition daily access to a mobile clinic staffed with a physician, who together with the nurses, refers unwell workers to the mill hospital for further evaluation (Supplement Table 2). Non-cutter manual agricultural workers are less frequently served by a mobile clinic. Indoor sugar factory workers have been supported by health promoters from November 2018 onward, and mechanized harvest workers from November 2019. During the study period, health promoters were increasingly trained to recognize early signs and symptoms of heat illness and to refer workers presenting with such symptoms to the ISA hospital for evaluation.

During the COVID-19 pandemic, workers had body temperature and oxygen saturation checked before, during, and after work shifts. Workers with signs or symptoms of COVID-19 were referred to the mill hospital for testing and contact tracing. Face masks were used, hands were washed, and sterilizing alcohol was provided before and after work shifts. Physical distancing was requested, and facilitated by using more buses for transport of workers to and from work.

### Ethical Permission

This study was approved by the Comité de Ética para Investigaciones Biomédicas (CEIB), Facultad de Ciencias Médicas, Universidad Nacional Autónoma de Nicaragua (UNAN-León), FWA000045231/IRB00003342. Data handling and statistical analyses carried out in Sweden were approved by the Swedish Ethical Review Authority (reg. no. 2023-01756-01). Data was collected as part of the company’s routine evaluation of health protection and care and subsequently shared with the researchers as secondary data, and thereby no consent was sought from workers on participation in research. We requested the clinical data anonymized, without information on name or hospital ID.

### Outcome Variable

Workers can seek care at the mill hospital at their own initiative, and by referral from health promoters or from the mobile clinic. The hospital is situated at the mill compound, is open 24 hours per day, all days of the year, and care is free of charge. Serum creatinine tests are routinely performed when workers seek care at the ISA hospital for any non-traumatic causes. Fischer and colleagues (2017) developed a passive kidney disease surveillance system at the ISA hospital, in which male workers with serum creatinine exceeding 1.3 mg/dl and female workers exceeding 1.1 mg/dl were considered to have acute kidney injury, and this case definition is used also in this analysis. A nurse employed by the mill OSH team asked workers fulfilling this definition about occupational and behavioral risk factors and symptoms (from a pre-specified list), use of antibiotics and non-steroidal anti-inflammatory drugs, and time of onset of symptoms (Fischer et al., 2017, 2018). This interview commonly took place within one day of hospital evaluation. The full extent of the laboratory examination performed (beyond serum creatinine) was at the discretion of the hospital physician. All laboratory analyses were performed at the hospital laboratory. Questionnaire and laboratory data were entered in a standardized format. The outcome events in the present study are such clinically diagnosed acute kidney injury (AKI-CD) between January 1, 2018, and May 1, 2023, prompted by workers seeking care due to symptoms or being referred from health promoters and mobile clinics due to clinical signs in the field.

### Exposure Variable

The exposure variable was constructed from data obtained from field-site WBGT measurements and a weather station at a central location at ISA, recording air temperature, humidity, solar radiation, and wind speed. The field-site WBGT measurements were performed by ISA employees, who place the WBGT monitors (QuestTemp34 [3M] or Kestrel 5400 Heat Stress Tracker [Nielsen-Kellerman]) in the open fields in a non-shaded location near where workers are working, following the workers to new locations each day. Field-site WBGT measurements were performed predominantly during harvest seasons (Supplement Figure 1). The activity performed by the workers monitored is recorded by the person performing the WBGT monitoring, and this was classified into activities performed among much and little green cane vegetation. WBGT was estimated from the weather station measurements using the Liljegren equation (Liljegren et al., 2008) implemented in the R package “wbgt” (Lieblich & Spector, 2017). A wind speed of 1.5 m/s was used if the wind speed was recorded to be lower than this, assuming that worker movements create air movement. Except for two periods of 46 and 3 days of incorrect solar radiation measurements during H2, weather station measurements were collected hourly on all but a very few days. Field-site WBGT measurements were performed half-hourly or hourly during working hours. For days with either no valid weather station data or no field-site WBGT measurements, only data from the available data source was used. Days with no data from field-site measurements or the weather station were considered to have missing data and were excluded from further analysis.

We utilized all the available data to predict noon WBGT based on all the available measurements from 6 am to 2 pm (reflecting the typical workday at ISA). We chose to predict noon WBGT because the source and quantity of heat data differed between days over the entire year, and the types of measurements (weather station/field), instruments, and frequency and temporal distribution of measurements throughout the day varied. By modeling the noon temperature using an approach that accounted for factors related to differences in the obtained heat measurements, systematic differences in measurements could be better accounted for than a simple mean or maximum WBGT each day. A mixed linear regression model with covariates for the time (half-hour) of the measurement, the type of measurement (field-site WBGT measurement, or WBGT estimated based on ISA weather station data), and the typical environment around the activity performed by the workers during the measurement (substantial green cane vegetation and no or very little green cane vegetation) as fixed effects, and a random intercept for each date. The overall mean and the coefficient for noon were added to this daily intercept to predict the daily noon WBGT based on the available weather measurements. This predicted noon WBGT was utilized as the exposure variable and entered as a linear term in the time series regression model.

### Statistical Analysis

The number of AKI-CD per day was modeled using negative binomial regression with the *glm* Stata version 17 command. Temporal variation across the year was modeled using a categorical variable for months, and long-term temporal trends were assessed by including a variable for a year. We sought to model the long-term trend by year, to understand changes in AKI hospitalization with increasing RSH intervention efforts each year. Rather than the calendar year, we classified the harvest year as beginning and ending at the end of July each year, thus coinciding with the end of the harvest season. The total ISA workforce size each month was entered as a variable with a coefficient constrained to 1, thereby providing a denominator that is needed for comparing longitudinal trends in incidence. The association between AKI rates and heat was assumed to be unaffected by changes in denominator size, being unchanged over a short time frame (days).

More advanced ways of modeling yearly variation and long-term trends, such as splines (Bhaskaran et al., 2013), or nonlinear exposure effects, were not considered suitable considering the small (*N* = 527) data size which does not allow for many parameters. Also, due to the activities and recruitment waves throughout the agricultural year which recur in the same months each year (e.g., harvest season), there are recurring step changes in the underlying worker population size, making the smooth transitions between seasons offered by splines or Fourier series unsuitable.

Daily noon WBGT was entered using a linear term, with a lag of up to 10 days. However, Day 8 to 10 lags did not display any association with AKI-CD and were not retained in the model. After inspection of the non-constrained lagged effect coefficients, lagged effects were constrained so that lag Days 2-4 and 5-7 had the same coefficients to reduce the multicollinearity of lagged effects and the number of model parameters (Bhaskaran et al., 2013). Lag Days 0 and 1 had unique coefficients. As the lag effects displayed signs of the “harvesting” phenomenon described by Bhaskaran et al. (2013), we estimated the net overall effect and its 95% confidence interval of heat over 0 to 7 lag days by summarizing each lag day effect over 1,000 iterations per day. A harvesting phenomenon is when a protective effect appears after a few lag days due to the pool of imminent cases (persons susceptible to the outcome) temporarily decreasing after a high-exposure day during which many suffer the event of interest. As a sensitivity analysis, we excluded all AKI-CD cases reporting that symptoms started more than one day before hospital diagnosis (*N*=266).

The noon WBGT long-term temporal trend was modeled using the same mixed linear regression described in the “Exposure variable” section, including also fixed effects for the season (week of the year) and harvest season (H1-6).

Data on AKI-CD demographical, occupational, behavioral, biochemical, and urine microscopy characteristics were summarized using percentages or medians with interquartile range (IQR). Changes in the reporting of symptoms during the study period were analyzed using logistic regression, with harvest included as a continuous variable.

## Results

There were a total of 527 workers presenting with AKI-CD during the 5.3 years of observation. Most patients were male (95%) and worked around 8 hours/day, 6 days/week in the field (Table 1). Clinical diagnosis of AKI most often occurred on Mondays-Fridays, with 75% of workers seeking care within 6 days from symptoms onset. Symptoms reported included headache, nausea, lumbar pain, dyspnea, and fever being reported by the majority. Only 5% reported NSAID intake.

**Table 1.** Demographic, Occupational and Behavioral Characteristics, and Symptoms of AKI-CD Cases (n = 527)

Variable	Men		Women	
	Respondents	Median (IQR) or *N* (%)	Respondents	Median (IQR) or *N* (%)
Total	500		27	
Age^a^ (years)	500	28 (24-35)	27	34 (30-45)
Occupational				
Field workers^a^ (%)	500	440 (88%)	27	25 (93%)
Hours worked/day^a^	372	8 (8-12)	23	8 (8-12)
Days worked/week^a^	372	6 (6-6)	23	6 (6-6)
**Day of week of clinical diagnosis**				
Sunday	500	26 (5%)	27	0
Monday	500	74 (15%)	27	7 (26%)
Tuesday	500	104 (21%)	27	5 (19%)
Wednesday	500	88 (18%)	27	5 (19%)
Thursday	500	90 (18%)	27	7 (26%)
Friday	500	81 (16%)	27	3 (11%)
Saturday	500	37 (7%)	27	0
**Behavior** ^a^				
Days since symptoms onset	434	2 (0-6)	26	2 (1-5)
NSAID use	500	25 (5%)	27	2 (7%)
Antibiotics use	500	25 (5%)	27	4 (15%)
**Symptoms** ^a^				
Headache	500	282 (56%)	27	19 (70%)
Nausea	500	276 (54%)	27	22 (81%)
Lumbar pain	500	263 (53%)	27	17 (63%)
Dyspnea	500	254 (51%)	27	15 (56%)
Fever	500	253 (51%)	27	13 (48%)
Loss of appetite	500	240 (48%)	27	16 (59%)
Muscle weakness	500	238 (48%)	27	17 (63%)
Vomiting	500	230 (46%)	27	18 (67%)
Paresthesia	500	228 (46%)	27	18 (67%)
Cramps	500	209 (42%)	27	17 (63%)
Myalgia	500	185 (37%)	27	11 (41%)
Fatigue	500	166 (34%)	27	12 (44%)
Joint pain	500	161 (33%)	27	14 (52%)
Abdominal pain	500	158 (32%)	27	16 (59%)
Dizziness	500	147 (29%)	27	14 (52%)
Tremor	500	142 (28%)	27	15 (56%)
Neck pain	499	111 (22%)	27	13 (48%)
Blurred vision	500	100 (20%)	27	11 (41%)
Dysuria	500	95 (19%)	27	11 (41%)
Cough	500	94 (19%)	27	11 (41%)
Chest pain	500	84 (17%)	27	9 (33%)
Confusion	500	46 (9%)	27	7 (26%)
Diarrhea	500	44 (9%)	27	3 (11%)
Rash	500	40 (8%)	27	4 (15%)
Edema	500	23 (5%)	27	12 (44%)

a Responses to a questionnaire administered by mill staff at hospital.

AKI-CD cases generally had elevated serum uric acid and creatine phosphokinase, and decreased potassium, calcium, and magnesium levels (Table 2). Markers of systemic inflammation (neutrophil: lymphocyte ratio [NLR], C-reactive protein [CRP], and erythrocyte sedimentation rate [ESR]) were generally elevated. The magnitude of elevation of these different markers of systemic inflammation differed by time between symptom onset and evaluation at the hospital (Supplement Figure 2). Leukocyturia was common, >80% with >5 leukocytes/visual field on inspection. Only two urine samples were nitrite-positive, indicating gram-negative bacterial urinary infections were largely absent, whereas non-hyaline casts were common, indicating kidney inflammation and injury were present.

**Table 2.** Biochemical and Urine Microscopy Findings in AKI-CD Cases (N = 527)

Variable	Reference interval^a^	Examined men, *N*	Median (IQR) or *N* (%)	Examined women, *N*	Median (IQR) or *N* (%)
**Serum examination**					
Creatinine (mg/dl)	0.9-1.3	500	2.0 (1.8-2.5)	27	1.7 (1.4-2.0)
eGFR (mL/min/1.73 m^2^)	>90	500	42 (34-51)	27	38 (33-49)
Urea (mg/dL)	10-50	198	42 (32-53)	10	31 (24-35)
Uric acid (mg/dL)	2.4-7.0	391	7.8 (6.7-9.0)	17	6.8 (5.7-7.8)
Sodium (mmol/L)	135-145	487	137 (135-139)	26	138 (136-140)
Potassium (mmol/L)	3.5-5.5	490	3.7 (3.2-4.0)	27	3.6 (3.2-4.2)
Ionized calcium (mg/dL)	1.15-1.35	463	1.18 (1.13-1.23)	24	1.20 (1.14-1.22)
Magnesium (mg/dL)	1.92-2.5	427	1.8 (1.5-2.0)	23	1.7 (1.4-2.1)
C-reactive protein (mg/L)	<6	159	24 (8-48)	10	51 (21-69)
Creatine phosphokinase (u/L)	24-190	415	220 (148-331)	23	137 (87-209)
**Blood examination**					
Hemoglobin (g/dL)	11-18	445	12.8 (11.8-13.7)	22	11.6 (10.5-12.3)
Hematocrit (%)	36-50	452	37 (34-40)	23	33 (31-37)
Leukocytes (N/mm^3^)	4500-11000	448	11950 (9200-15625)	22	9700 (7800-12400)
Neutrophils (N/mm^3^)		448	8960 (6240-13000)	22	6780 (5400-9420)
Neutrophils (% of leukocytes)	52-67% of leukocytes	448	76 (68-73)	22	73 (68-82)
Lymphocytes (N/mm^3^)		448	2540 (1940-3200)	22	2200 (1800-2860)
Lymphocytes (% of leukocytes)	21-35% of leukocytes	448	22 (15-30)	22	25 (18-32)
Neutrophil-Lymphocyte ratio	2.84^b^	448	3.5 (2.3-5.7)	22	3.0 (2.1-4.6)
Thrombocytes (1000/mm^3^)	150-500	443	261 (227-307)	22	279 (246-315)
Erythrocyte sedimentation rate (mm/hr)	<10	121	25 (10-43)	10	35 (18-40)
**Urine examination**					
Non-transparent on inspection		439	313 (72%)	27	26 (96%)
Dipstick					
Density	1005-1030	441	1020 (1015-1020)	27	1015 (1010-1020)
pH	5-9	440	6 (5-6.5)	27	6 (5-6)
Proteinuria (>trace)		439	195 (44%)	27	7 (30%)
Hematuria (any +)		440	149 (34%)	27	11 (41%)
Glucosuria (any +)		440	51 (12%)	27	2 (7%)
Nitrite (any +)		440	1 (0%)	26	1 (4%)
Ketonuria (any +)		440	33 (8%)	27	1 (4%)
Microscopy					
Leukocytes > 5/field		440	352 (80%)	27	25 (93%)
Leukocytes >50/field or uncountable		440	164 (37%)	27	11 (41%)
Erythrocytes > 5/field		440	101 (23%)	27	10 (37%)
Any non-hyaline casts	Absence	440	167 (39%)	27	9 (33%)
Amorphous urate crystals	Absence	440	188 (43%)	27	14 (54%)
Amorphous phosphate crystals	Absence	440	33 (8%)	27	1 (14%)
Amorphous oxalate crystals	Absence	440	5 (1%)	27	0 (0%)

a Reference values are from ISA hospital laboratory. ^b^ 97.5^th^ percentile among pre-employment screening visits during 2018-2019 harvest (Manuscript in process).

**Figure 1.**
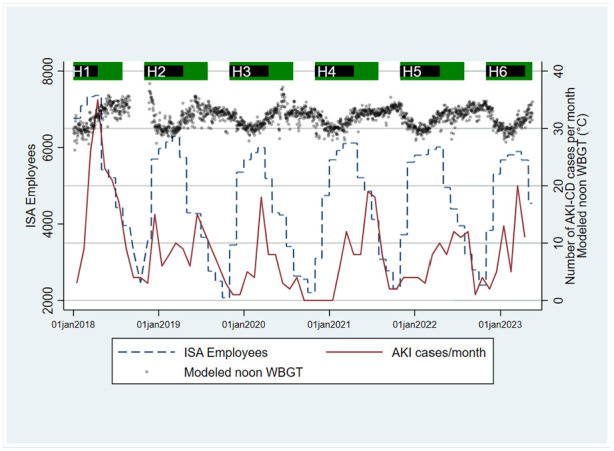
Number of employees, hospital-diagnosed acute kidney injury (AKI-CD) cases per month and modeled noon WBGT at Ingenio San Antonio (ISA), Nicaragua.

**Figure 2.**
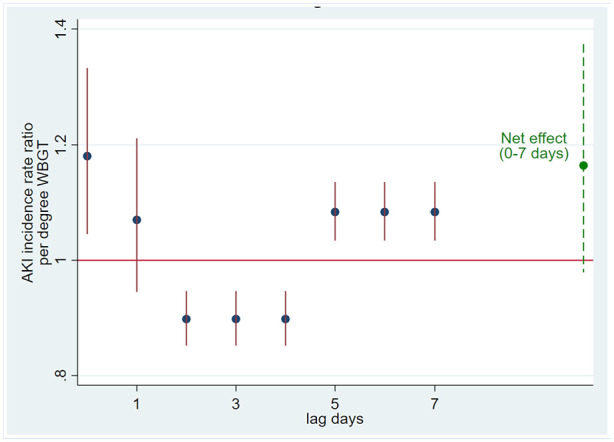
Association between environmental heat (wet bulb globe temperature) increase and hospital-evaluated acute kidney injury incidence ratio at Ingenio San Antonio, Nicaragua.

The number of AKI-CD cases varied throughout the year, typically peaking during the harvest season in January-June, when the number of employed workers was the highest, whereas WBGT had the highest levels during March-September (Figure 1). Noon WBGT varied between approximately 29°C and 34°C throughout the year. AKI-CD case numbers were generally low in November-December, a period of acclimatization for new and returning harvest workers characterized by lower production targets and also somewhat lower temperatures than in later parts of the harvest. Same-day WBGT was positively associated with a higher incidence of AKI-CD, increasing by 18%/°C (95% CI [4, 33%]). The association was inverse on lag days 2-4 but resumed a positive association during lag days 5-7 (Figure 2). The overall association on lag days 0-7 indicated a 14% increase in AKI-CD incidence per 1°C WBGT (95% CI [−5%, 37%]). A similar pattern remained after excluding AKI-CD cases reporting the onset of symptoms more than one day before clinical diagnosis.

**Figure 3.**
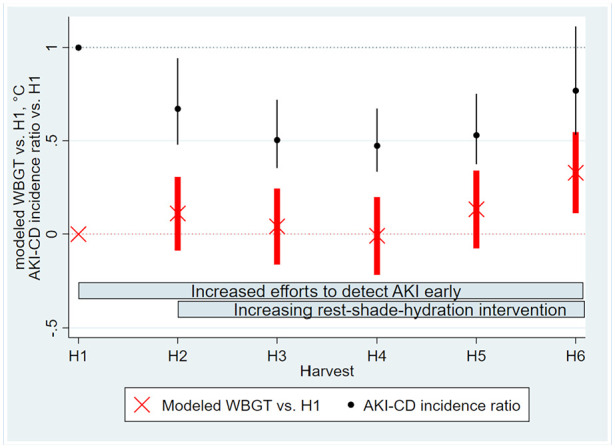
Trends in heat and hospital-evaluated acute kidney injury during the study period. Bars denote 95% confidence intervals, black denoting AKI-CD incidence ratio and red the modeled noon WBGT.

The AKI-CD incidence decreased during the study period, however plateauing or beginning to reverse from H3-H4 onwards (Figure 3). There was no obvious linear trend in modeled noon WBGT during the study, but H6 was significantly hotter than H1 (Figure 3). Each year (except for H5 to H6), AKI-CD patients generally had a higher eGFR (Supplement Figure 4), sought care earlier (Supplement Figure 5), and reported fewer symptoms (Supplement Table 3) at the time of AKI diagnosis.

## Discussion

The environmental heat stress, measured as WBGT, seemed to have both an immediate effect on the sugarcane workers, leading to a higher incidence of AKI-CD on the same and subsequent day, and a more prolonged effect lasting up to 1 week. With noon WBGT levels typically exceeding 30°C (86°F) for a large proportion of the year, the workers in the present study are exposed to significant environmental heat stress (American Conference of Governmental Industrial Hygienists, 2019). The peak in the number of AKI-CD cases in January-June rather than the hottest months March to September can likely be explained by a larger workforce employed in the physically demanding sugarcane harvest during these months.

The apparent inverse effect of heat over 2 to 4 lag days can likely be explained by “harvesting” (Bhaskaran et al., 2013), that is, workers who would otherwise fall ill with AKI-CD sooner or later are pushed into hospital care earlier due to heat, thereby reducing the pool of imminent AKI-CD cases. Consistent with our findings of a lagged effect of environmental heat of up to 1 week, the workers often reported that symptoms started a few days before clinical diagnosis. Findings of the timing of symptoms onset in relation to markers of systemic inflammation are consistent with the expected trajectory of these inflammation markers; NLR can increase within hours, CRP within days and ESR within a week. The pathophysiological, inflammatory, process leading up to a clinical diagnosis of kidney injury likely started several days prior, consistent with the second peak in heat effects at lag days 5 to 7.

This study again identifies an association between heat and kidney injury, this time focusing on the external component of heat stress rather than metabolic heat production as in previous studies (Glaser et al., 2020; Hansson et al., 2019). The long-term prognosis of this injury remains unknown, although Fischer et al. (2017, 2018) reported that 8% of AKI-CD cases from ISA had developed CKDnt within months of the injury event. In future research, we will aim to follow up the patients in the current study to determine the long-term effects of these injury events.

Notably, workers sought care earlier, with fewer symptoms and with less pronounced kidney dysfunction each year of the study, except for H5 to H6. We ascribe this to an increased awareness that parallels the enhanced RSH intervention. It is therefore likely that a substantial proportion of AKI-CD events in later parts of the study would not have been clinically diagnosed in earlier parts of the study. Previous research in this population highlights that a large proportion of active workers have biochemical evidence of kidney injury, report similar symptoms as the AKI-CD cases in this study, and continue working (Glaser et al., 2020; Hansson et al., 2019, 2021), indicating that some workers might continue working despite suffering from kidney injury but that this proportion may have decreased. This increased awareness of symptoms indicative of kidney injury and healthcare seeking among workers is beneficial, as it can enable earlier diagnosis and individual-level prevention. Despite this improved case detection, and the potential for a positive referral bias, AKI-CD incidence decreased as the RSH intervention was enhanced, indicating a preventive effect. Fischer et al. reported 246 AKI-CD cases in 2015 alone at the same hospital (Fischer et al., 2017), that is, almost half the number recorded over the 5.3 years observed in this study. Lower environmental temperatures are not a credible explanation for the reduced AKI-CD incidence considering stable or increasing WBGT during the study period (Figure 3). However, the significantly hotter H6 was associated with an increase in AKI-CD incidence (Figure 3).

This study encompasses all ISA workers, including both workers experiencing the RSH intervention that was monitored and evaluated as part of the Adelante Initiative (Glaser et al., 2020, 2022) and a larger number of workers not included in that specific monitoring and evaluation program but still receiving enhanced RSH interventions. A larger decline in AKI has been reported within the job groups included in the Adelante Initiative (94% from H1 to H3; Glaser et al., 2022) than in this study of the overall ISA workforce, consistent with a larger effect of a closely monitored RSH intervention.

The high proportion of male cases (95%) is similar to the 89.5% previously reported from the mill (Fischer et al., 2017). In all, 15% of ISA’s workforce is female, possibly suggesting an excess AKI-CD risk for male workers. There is also a clear disproportionate male preponderance among asymptomatic seed cutters with measured increases in serum creatinine (Glaser et al., 2020). More men engaging in heavy work in hot environments is likely the main explanation for this gender difference, but biological sex differences may also play a role. NSAID consumption does not explain the high incidence of AKI-CD in this population, being reported by only 5% of the patients.

The probable effect magnitude at 14% increase/WBGT °C (95% CI [−5%, 37%]) over the past week is much larger than the 1% risk increase/ambient temperature °C seen in many general populations (Liu et al., 2021). This is most likely explained by the high metabolic workload of the sugarcane workers (Lucas et al., 2023). A high metabolic heat production in a high environmental heat leads to a substantially larger heat stress than low metabolic heat production in the same environmental heat. Moreover, WBGT is likely a better heat stress indicator than ambient temperature. Importantly, this study reflects the association between heat and AKI-CD at a large agro-industrial enterprise working consciously with heat stress prevention (Pacheco-Zenteno et al., 2021). The association between heat and kidney injury is likely stronger among workers with similar metabolic workloads and environmental heat stress but where heat prevention efforts are weaker or non-existent.

This study provides an example of how data can and should be used by employers in low- and middle-income settings to monitor occupational health, understand causes of occupationally linked disease, and evaluate preventive efforts. Trained local health and safety personnel implemented the RSH intervention, educated workers on heat illness and monitored them for signs of such, and collected the data necessary for intervention evaluation. Having a hospital integrated into the occupational health system was also key to conducting this study. However, routinely collected clinical data at the physician’s discretion is susceptible to missing data, such as seen for some biochemical results in this study, and this is a limitation affecting especially the analysis of inflammation biomarkers. Information on job type was not collected with sufficient granularity to allow for analysis of which jobs are at high risk.

Environment and outcome data collection by non-research staff enabled data collection to continue during the COVID-19 pandemic. Although production continued during the COVID-19 pandemic, field conditions were still affected. Research staff were temporarily unable to evaluate intervention implementation via field visits and were not allowed to visit the hospital, which is a limitation.

## Conclusion/Implications to Occupational Health Practice

The study described the association between environmental heat stress and acute kidney disease among highly physically active workers in a CKDnt hotspot, strengthening the case for occupational heat stress as an important determinant of CKDnt initiation and progression. Kidney injury events became less common and less severe as heat stress prevention through an RSH intervention was enhanced and efforts were made for early diagnosis of heat-induced kidney injury, but these events did not disappear and partly reversed in H6, the hottest harvest during the study period. The association between AKI-CD and external heat highlights the need to adapt daily operations depending on heat levels, and for employers to have contingency plans in case of forecasted heat waves. In some climates and types of work, WBGT measurements or app-based predictions of WBGT (e.g., ClimApp [Kingma et al., 2021]) may enable rapid adaptation of rest-work schedules, whereas seasonally varying rest-work schedules may be more feasible in other settings.

**Applying Research to Occupational Health Practice**

This work highlights the importance of comprehensive occupational health and safety protocols promoting water, rest, and shade for heat-exposed workers. Such interventions should include trained occupational health and safety promoters, and nurses are a key healthcare profession that can be trained to help recognize and prevent acute kidney injury and its sequelae in the workplace. Water, rest, and shade interventions should also adapt over time to provide optimal protection to and meet the needs of workers in a warming climate. Contingency plans may also be necessary to protect workers during periods of acute weather events, such as heatwaves.

## Supplemental Material

sj-docx-1-whs-10.1177_21650799241235410 – Supplemental material for Association Between Acute Kidney Injury Hospital Visits and Environmental Heat Stress at a Nicaraguan Sugarcane Plantation Supplemental material, sj-docx-1-whs-10.1177_21650799241235410 for Association Between Acute Kidney Injury Hospital Visits and Environmental Heat Stress at a Nicaraguan Sugarcane Plantation by Erik Hansson, Kristina Jakobsson, Jason R. Glaser, Catharina Wesseling, Denis Chavarría, Rebekah A. I. Lucas and David H. Wegman in Workplace Health & Safety

## References

[bibr1-21650799241235410] American Conference of Governmental Industrial Hygienists. (2019). Threshold limit values (TLVs) and Biological Exposure Index (BEIs) for chemical substances and physical agents.

[bibr2-21650799241235410] Bhaskaran K. Gasparrini A. Hajat S. Smeeth L. Armstrong B. (2013). Time series regression studies in environmental epidemiology. International Journal of Epidemiology, 42(4), 1187–1195. 10.1093/ije/dyt092 23760528 PMC3780998

[bibr3-21650799241235410] Bodin T. Garcia-Trabanino R. Weiss I. Jarquin E. Glaser J. Jakobsson K. Lucas R. A. Wesseling C. Hogstedt C. Wegman D. H. (2016). Intervention to reduce heat stress and improve efficiency among sugarcane workers in El Salvador: Phase 1. Occupational and Environmental Medicine, 73(6), 409–416. 10.1136/oemed-2016-103555 27073211 PMC4893112

[bibr4-21650799241235410] Budd G. M. (2008). Wet-bulb globe temperature (WBGT); its history and its limitations. Journal of Science and Medicine in Sport, 11(1), 20–32. 10.1016/j.jsams.2007.07.003 17765661

[bibr5-21650799241235410] Butler-Dawson J. Krisher L. Yoder H. Dally M. Sorensen C. Johnson R. J. Asensio C. Cruz A. Johnson E. C. Carlton E. J. Tenney L. Asturias E. J. Newman L. S. (2019). Evaluation of heat stress and cumulative incidence of acute kidney injury in sugarcane workers in Guatemala. International Archives of Occupational and Environmental Health, 92(7), 977–990. 10.1007/s00420-019-01426-3 30997573 PMC6768910

[bibr6-21650799241235410] Chapman C. L. Johnson B. D. Vargas N. T. Hostler D. Parker M. D. Schlader Z. J. (2020). Hyperthermia and dehydration during physical work in the heat both contribute to the risk of acute kidney injury. Journal of Applied Physiology, 128, 715–728. 10.1152/japplphysiol.00787.2019 32078468 PMC7191500

[bibr7-21650799241235410] Fischer R. S. B. Mandayam S. Chavarria D. Vangala C. Nolan M. S. Garcia L. L. Palma L. Garcia F. Garcia-Trabanino R. Murray K. O. (2017). Clinical evidence of acute mesoamerican nephropathy. American Journal of Tropical Medicine and Hygiene, 97(4), 1247–1256. 10.4269/ajtmh.17-0260 28722618 PMC5637619

[bibr8-21650799241235410] Fischer R. S. B. Vangala C. Mandayam S. Chavarria D. Garcia-Trabanino R. Garcia F. Garcia L. L. Murray K. O. (2018). Clinical markers to predict progression from acute to chronic kidney disease in Mesoamerican nephropathy. Kidney International, 94(6), 1205–1216. 10.1016/j.kint.2018.08.020 30466566 PMC8719352

[bibr9-21650799241235410] Glaser J. Hansson E. Weiss I. Wesseling C. Jakobsson K. Ekstrom U. Apelqvist J. Lucas R. A. Arias Monge E. Peraza S. Hogstedt C. Wegman D. H. (2020). Preventing kidney injury among sugarcane workers: Promising evidence from enhanced workplace interventions. Occupational and Environmental Medicine, 77(8), 527–534. 10.1136/oemed-2020-106406 32404530 PMC7402461

[bibr10-21650799241235410] Glaser J. Wegman D. H. Arias-Monge E. Pacheco-Zenteno F. Prince H. Chavarria D. Martinez-Cuadra W. J. Jakobsson K. Lucas R. A. Weiss I. Wesseling C. (2022). Workplace intervention for heat stress: Essential elements of design, implementation, and assessment. International Journal of Environmental Research and Public Health, 19(7), Article 3779. 10.3390/ijerph19073779 PMC8998134 35409463

[bibr11-21650799241235410] Hansson E. Glaser J. Jakobsson K. Weiss I. Wesseling C. Lucas R. A. I. Wei J. L. K. Wegman D. H. (2020). Pathophysiological mechanisms by which heat stress potentially induces kidney inflammation and chronic kidney disease in sugarcane workers. Nutrients, 12, Article 1639. 10.3390/nu12061639 PMC7352879 32498242

[bibr12-21650799241235410] Hansson E. Glaser J. Weiss I. Ekström U. Apelqvist J. Hogstedt C. Peraza S. Lucas R. A. Jakobsson K. Wesseling C. Wegman D. H. (2019). Workload and cross-harvest kidney injury in a Nicaraguan sugarcane worker cohort. Occupational and Environmental Medicine, 76(11), 818–826. 10.1136/oemed-2019-105986 31611303 PMC6839725

[bibr13-21650799241235410] Hansson E. Mansourian A. Farnaghi M. Petzold M. Jakobsson K. (2021). An ecological study of chronic kidney disease in five Mesoamerican countries: Associations with crop and heat. BMC Public Health, 21(1), Article 840. 10.1186/s12889-021-10822-9 PMC8088703 33933045

[bibr14-21650799241235410] Kingma B. R. M. Steenhoff H. Toftum J. Daanen H. A. M. Folkerts M. A. Gerrett N. Gao C. Kuklane K. Petersson J. Halder A. Zuurbier M. Garland S. W. Nybo L. (2021). ClimApp—Integrating personal factors with weather forecasts for individualised warning and guidance on thermal stress. International Journal of Environmental Research and Public Health, 18(21), Article 11317. 10.3390/ijerph182111317 PMC8583482 34769832

[bibr15-21650799241235410] Kupferman J. Ramirez-Rubio O. Amador J. J. Lopez-Pilarte D. Wilker E. H. Laws R. L. Sennett C. Robles N. V. Lau J. L. Salinas A. J. Kaufman J. S. Weiner D. E. Scammel M. K. McClean M. D. Brooks D. R. (2018). Acute kidney injury in sugarcane workers at risk for mesoamerican nephropathy. American Journal of Kidney Diseases, 72(4), 475–482. 10.1053/j.ajkd.2018.04.014 30042041

[bibr16-21650799241235410] Lieblich M. Spector J. (2017). _wbgt: Wet bulb globe temperature_ (R package version 1.2). https://github.com/mdljts/wbgt/

[bibr17-21650799241235410] Liljegren J. C. Carhart R. A. Lawday P. Tschopp S. Sharp R. (2008). Modeling the wet bulb globe temperature using standard meteorological measurements. Journal of Occupational and Environmental Hygiene, 5(10), 645–655. 10.1080/15459620802310770 18668404

[bibr18-21650799241235410] Liu J. Varghese B. M. Hansen A. Borg M. A. Zhang Y. Driscoll T. Morgan G. Dear K. Gourley M. Capon A. Bi P. (2021). Hot weather as a risk factor for kidney disease outcomes: A systematic review and meta-analysis of epidemiological evidence. Science of the Total Environment, 801, Article 149806. 10.1016/j.scitotenv.2021.149806 34467930

[bibr19-21650799241235410] Lucas R. A. I. Skinner B. D. Arias-Monge E. Jakobsson K. Wesseling C. Weiss I. Poveda S. Cerda-Granados F. Glaser J. Hansson E. Wegman D. H. (2023). Targeting workload to ameliorate risk of heat stress in industrial sugarcane workers. Scandinavian Journal of Work, Environment and Health, 49(1), 43–52. 10.5271/sjweh.4057 PMC10549916 36209512

[bibr20-21650799241235410] McClean M. D. Laws R. L. Ramirez Rubio O. Brooks D. R. (2010). Industrial Hygiene/Occupational Health Assessment: Evaluating potential hazards associated with chemicals and work practices at the Ingenio San Antonio (Chichigalpa, Nicaragua). https://www.saltra.una.ac.cr/images/SALTRA/Documentacion/MeN/FINALIHReport-AUG302010-ENGLISH.pdf

[bibr21-21650799241235410] Occupational Safety and Health Administration. (2022). National emphasis program–Outdoor and indoor heat-related hazards. https://www.osha.gov/sites/default/files/enforcement/directives/CPL_03-00-024.pdf

[bibr22-21650799241235410] Pacheco-Zenteno F. Glaser J. Jakobsson K. Weiss I. Arias-Monge E. Gyllensten K. (2021). The prevention of occupational heat stress in sugarcane workers in Nicaragua—An interpretative phenomenological analysis. Frontiers in Public Health, 9, Article 713711. 10.3389/fpubh.2021.713711 PMC8545795 34712636

[bibr23-21650799241235410] Pan American Health Organization. (2017). Epidemic of chronic kidney disease in agricultural communities in Central America. Case Definitions, Methodological Basis and Approaches for Public Health Surveillance [Publications]. https://iris.paho.org/handle/10665.2/34132

[bibr24-21650799241235410] Sorensen C. J. Butler-Dawson J. Dally M. Krisher L. Griffin B. R. Johnson R. J. Lemery J. Asensio C. Tenney L. Newman L. S. (2019). Risk factors and mechanisms underlying cross-shift decline in kidney function in Guatemalan sugarcane workers. Journal of Occupational and Environmental Medicine, 61(3), 239–250. 10.1097/jom.0000000000001529 30575695 PMC6416034

[bibr25-21650799241235410] Wegman D. H. Apelqvist J. Bottai M. Ekström U. García-Trabanino R. Glaser J. Hogstedt C. Jakobsson K. Jarquin E. Lucas R. A. Weiss I. Wesseling C. Bodin T. (2018). Intervention to diminish dehydration and kidney damage among sugarcane workers. Scandinavian Journal of Work, Environment and Health, 44(1), 16–24. 10.5271/sjweh.3659 28691728

[bibr26-21650799241235410] Wesseling C. Glaser J. Rodríguez-Guzmán J. Weiss I. Lucas R. Peraza S. da Silva A. S. Hansson E. Johnson R. Hogstedt C. Wegman D. H. Jakobsson K. (2020). Chronic kidney disease of non-traditional origin in Mesoamerica: A disease primarily driven by occupational heat stress. Revista Panamericana de Salud Publica, 44, Article e15. 10.26633/RPSP.2020.15 PMC6984407 31998376

[bibr27-21650799241235410] Yaglou C. P. Minard D. (1957). Control of heat casualties at military training centers. AMA Archives of Industrial Health, 16(4), 302–316. 13457450

