# Supplementary material for: Association Between Acute Kidney Injury Hospital Visits and Environmental Heat Stress at a Nicaraguan Sugarcane Plantation
Source: Workplace Health Saf. 2024 Apr 9;72(4):131–42. doi: 10.1177/21650799241235410 (PMC11055406; doi:10.1177/21650799241235410)
Supplement: sj-docx-1-whs-10.1177_21650799241235410 – Supplemental material for Association Between Acute Kidney Injury Hospital Visits and Environmental Heat Stress at a Nicaraguan Sugarcane Plantation [file sj-docx-1-whs-10.1177_21650799241235410.docx]

Supplement Table 1. Mandated rest periods (minutes) per hour at Ingenio San Antonio. H1=2017/2018 harvest season.

|  |  | 6:00 AM | 7:00 AM | 8:00 AM | 9:00 AM | 10:00 AM | 11:00 AM | 12:00 PM | 1:00 PM | 2:00 PM |
| --- | --- | --- | --- | --- | --- | --- | --- | --- | --- | --- |
| Burned cane cutters | H1 | Start |  |  | 20 | 10 | 20 | End |  |  |
|  | H2 | Start |  | 10 | 20 | 15 | 15 | End |  |  |
|  | H3 onwards | Start | 10 | 15 | 20 | 20 | 15 | End |  |  |
| Seed cutters | H1 | Start |  |  | 20 |  |  | 30 |  | End |
|  | H2 | Start |  | 10 | 10 | 10 | 10 | 30 |  | End |
|  | H3 onwards | Start | 10 | 15 | 20 | 20 | 15 | 30 | 20 | End |
| Other field workers | H1 | Start |  |  | 20 |  |  | 30 |  | End |
|  | H2 | Start |  | 10 | 10 | 10 | 10 | 30 |  | End |
|  | H3 onwards | Start | 5 | 10 | 10 | 10 | 10 | 30 | 20 | End |

Supplement Table 2. Causes of worker referral from field-based mobile clinic to hospital.

| **Referral from mobile clinic to hospital internal medicine emergency department** |
| --- |
| **Signs and symptoms of heat stress which do not stabilize after first aid measures, e.g:** |
| Headache |
| Dizziness |
| Lightheadedness/fainting |
| Nausea/vomiting |
| Elevated body temperature/fever |
| Low or very dark urine production |
| Weakness and fatigue |
| Muscle cramps |
| Rapid and weak pulse |
| Excessive or absence of sweating |
| Irritability, disorientation, confusion, agitation |
| Impaired consciousness |
| Convulsions |
| **Pronounced dehydration** |
| Weight loss >3% |
| **Abnormal field-side laboratory examinations in the mobile clinic (in dehydrated worker)** |
| Abundant leukocytes in urine |
| Amorphous uric acid crystals |

Supplement Table 3 Symptoms confirmed^1^ by AKI-CD cases, by harvest

|  | AKI-CD cases confirming symptom, N (%) | | | | | |  |
| --- | --- | --- | --- | --- | --- | --- | --- |
| Symptom | H1 | H2 | H3 | H4 | H5 | H6 | p (linear trend) |
| Headache | 90 (70%) | 69 (66%) | 39 (57%) | 27 (37%) | 32 (42%) | 44 (59%) | <0.001 |
| Nausea | 89 (69%) | 71 (68%) | 38 (55%) | 26 (36%) | 34 (44%) | 33 (44%) | <0.001 |
| Lumbar pain | 84 (65%) | 64 (62%) | 33 (48%) | 28 (38%) | 35 (45%) | 36 (48%) | <0.001 |
| Dyspnea | 93 (72%) | 56 (54%) | 38 (55%) | 18 (25%) | 26 (34%) | 38 (51%) | <0.001 |
| Fever | 85 (66%) | 71 (68%) | 36 (52%) | 17 (23%) | 23 (30%) | 34 (45%) | <0.001 |
| Loss of appetite | 95 (74%) | 68 (65%) | 26 (38%) | 16 (22%) | 21 (27%) | 30 (40%) | <0.001 |
| Muscle weakness | 89 (69%) | 69 (66%) | 32 (46%) | 18 (25%) | 22 (29%) | 25 (33%) | <0.001 |
| Vomiting | 70 (54%) | 57 (55%) | 28 (41%) | 31 (42%) | 35 (45%) | 27 (36%) | <0.001 |
| Paresthesia | 80 (62%) | 60 (58%) | 26 (38%) | 20 (27%) | 24 (31%) | 36 (48%) | <0.001 |
| Cramps | 66 (51%) | 50 (48%) | 17 (25%) | 29 (40%) | 35 (45%) | 29 (39%) | 0.09 |
| Myalgia | 63 (49%) | 47 (45%) | 23 (33%) | 8 (11%) | 21 (27%) | 34 (45%) | 0.01 |
| Fatigue | 66 (51%) | 44 (42%) | 22 (32%) | 15 (21%) | 14 (18%) | 17 (23%) | <0.001 |
| Joint pain | 68 (53%) | 46 (44%) | 20 (29%) | 10 (14%) | 12 (16%) | 19 (25%) | <0.001 |
| Abdominal pain | 60 (47%) | 48 (46%) | 23 (33%) | 9 (12%) | 14 (18%) | 20 (27%) | <0.001 |
| Dizziness | 67 (52%) | 38 (37%) | 19 (28%) | 8 (11%) | 13 (17%) | 16 (21%) | <0.001 |
| Tremor | 64 (50%) | 36 (35%) | 19 (28%) | 8 (11%) | 11 (14%) | 19 (25%) | <0.001 |
| Neck pain | 48 (37%) | 37 (36%) | 14 (20%) | 3 (4%) | 11 (14%) | 11 (15%) | <0.001 |
| Blurred vision | 49 (38%) | 29 (28%) | 11 (16%) | 2 (3%) | 11 (14%) | 9 (12%) | <0.001 |
| Dysuria | 48 (37%) | 32 (31%) | 10 (14%) | 1 (1%) | 7 (9%) | 8 (11%) | <0.001 |
| Cough | 40 (31%) | 31 (30%) | 15 (22%) | 4 (5%) | 7 (9%) | 8 (11%) | <0.001 |
| Chest pain | 37 (29%) | 26 (25%) | 11 (16%) | 1 (1%) | 6 (8%) | 12 (16%) | <0.001 |
| Confusion | 19 (15%) | 14 (13%) | 6 (9%) | 1 (1%) | 9 (12%) | 4 (5%) | 0.02 |
| Diarrhea | 13 (10%) | 17 (16%) | 5 (7%) | 1 (1%) | 3 (4%) | 8 (11%) | 0.09 |
| Rash | 20 (16%) | 12 (12%) | 5 (7%) | 1 (1%) | 1 (1%) | 5 (7%) | <0.001 |
| Edema | 17 (13%) | 7 (7%) | 4 (6%) | 1 (1%) | 1 (1%) | 5 (7%) | <0.001 |
|  |  |  |  |  |  |  |  |
| Number of symptoms reported, median (IQR) | 13 (7-17) | 11 (6-15) | 7 (2-13) | 3 (2-5) | 3 (2-5) | 6 (4-10) |  |

1. Responses to a questionnaire administered by trained nurses at the mill OSH department, typically in the day after hospital evaluation. Symptoms were asked based on a list of symptoms, responded by yes/no/unknown.


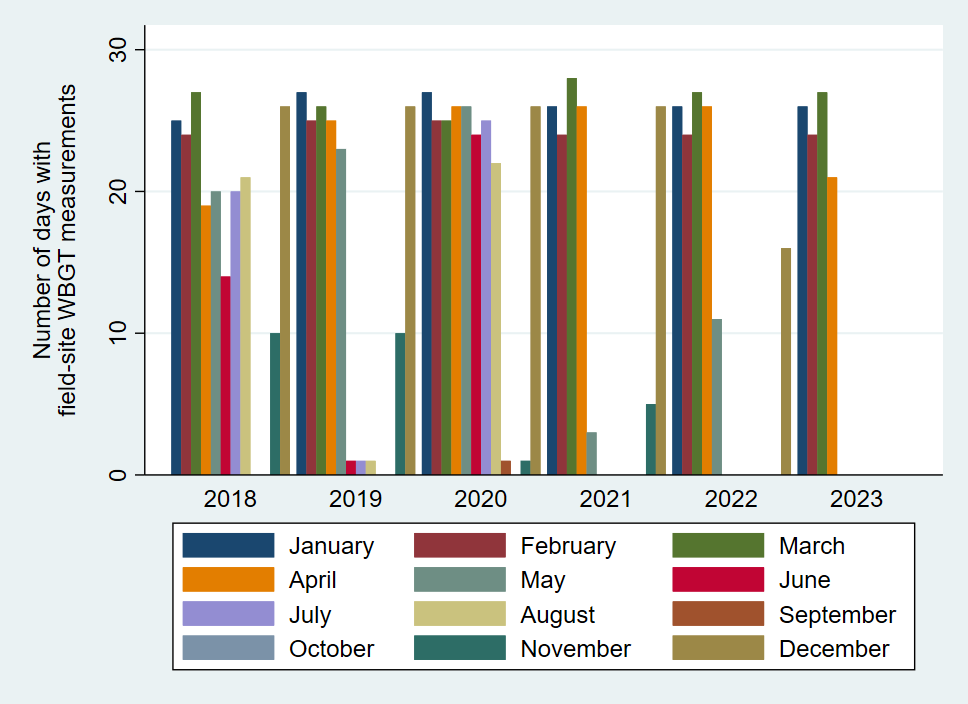


Supplement Figure 1. Days with field-site WBGT measurements by month.


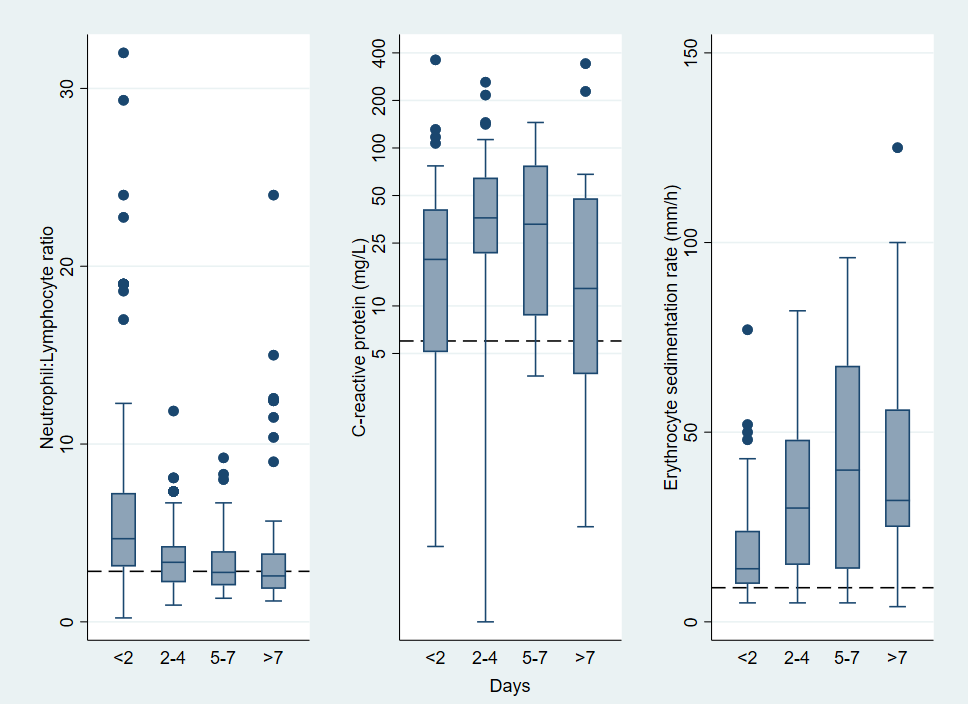


Supplement Figure 2. Distribution of biomarkers of systemic inflammation, by time between onset of symptoms and hospital evaluation


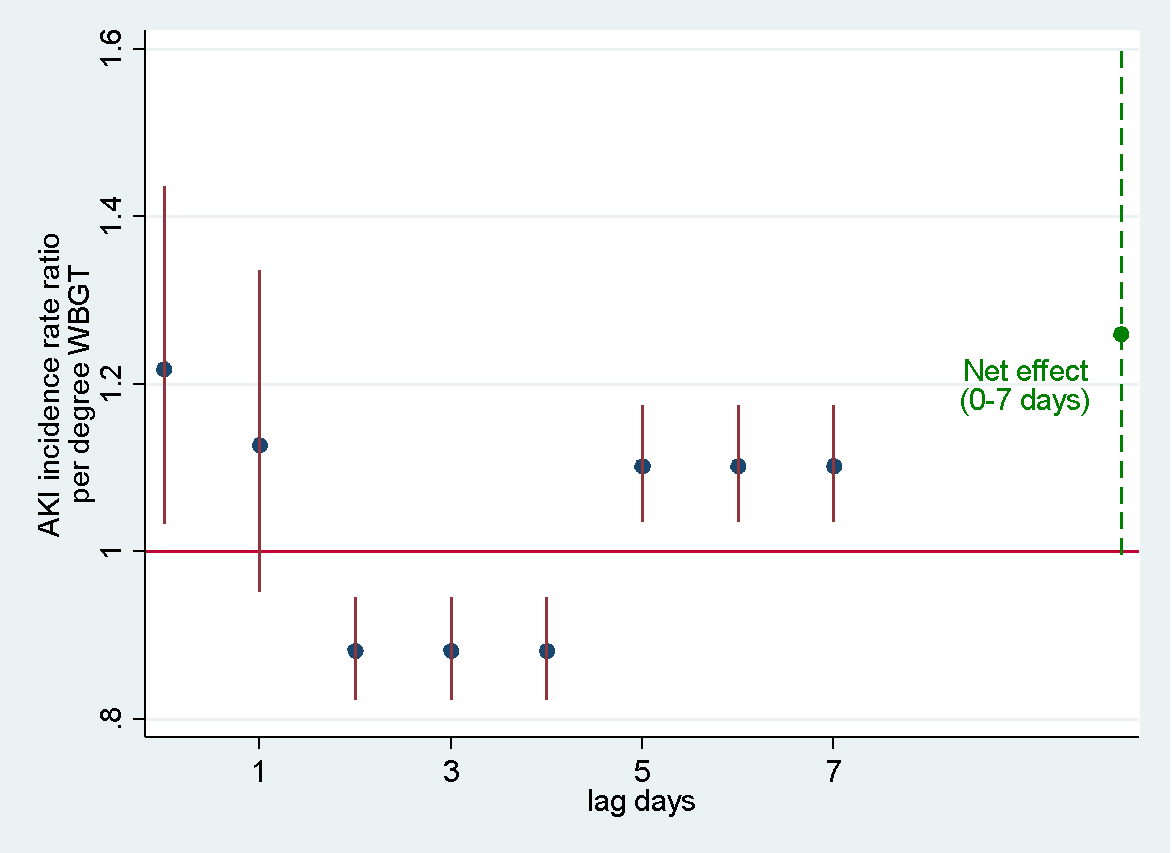


Supplement Figure 3. Association between environmental heat (wet bulb globe temperature) and hospital-evaluated acute kidney injury incidence ratio at Ingenio San Antonio, Nicaragua, restricted to workers reporting onset of symptoms on the same or preceding one day.


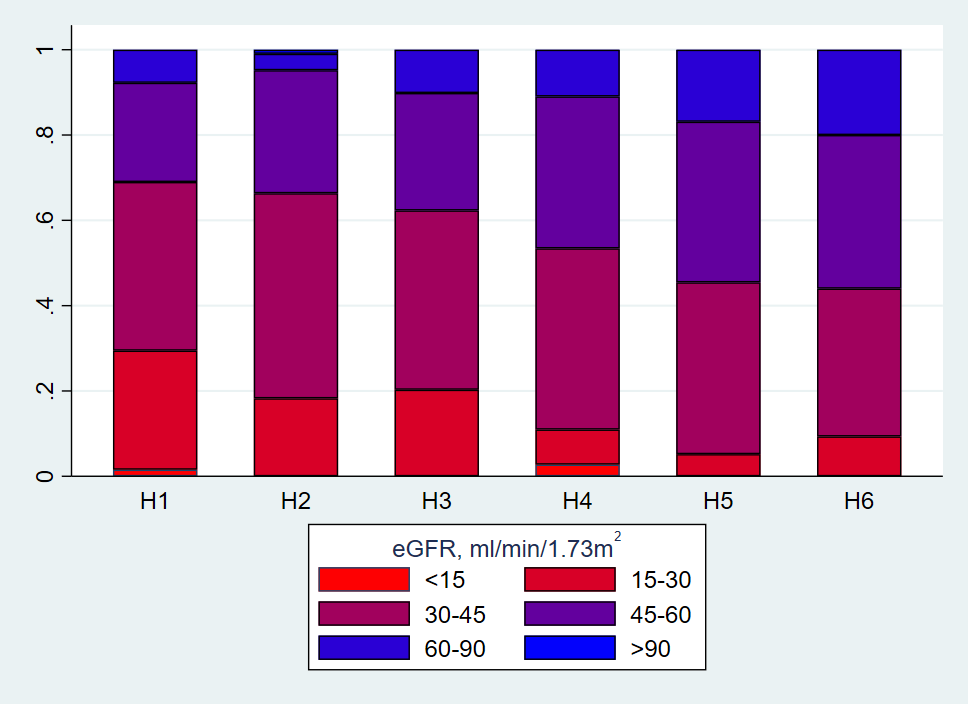


Supplement Figure 4. Distribution of estimated glomerular filtration rate (eGFR) among workers clinically diagnosed with acute kidney injury (AKI-CD) at Ingenio San Antonio harvests 1-6


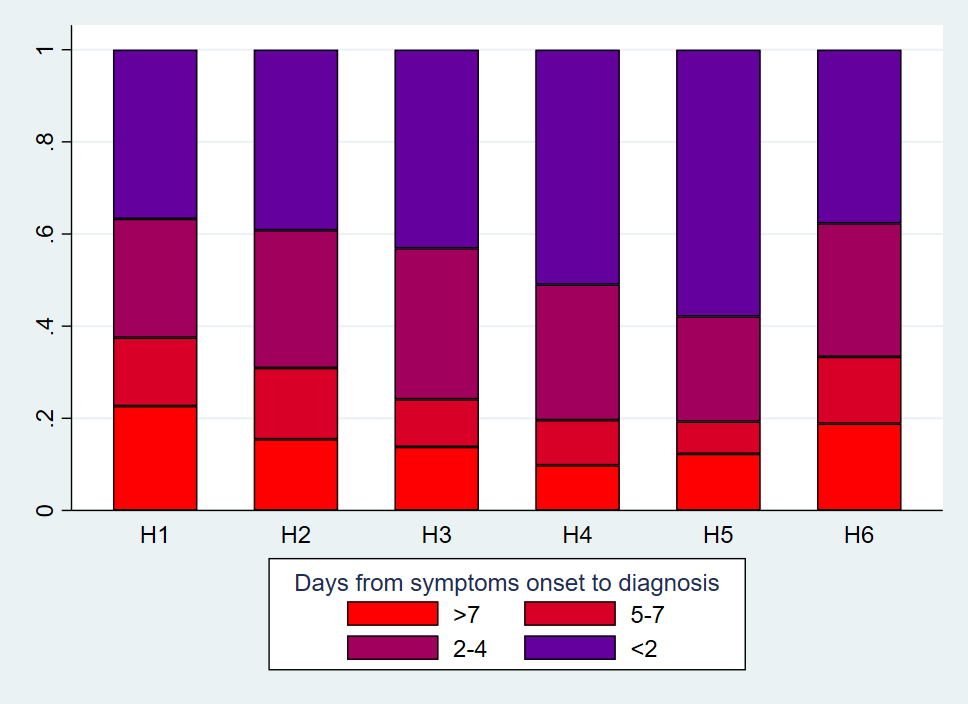


Supplement Figure 5. Distribution of days between symptoms onset and diagnosis among workers clinically diagnosed with acute kidney injury (AKI-CD) at Ingenio San Antonio harvests 1-6
